# Antigen/adjuvant‐free liposome induces adjuvant effects for enhancing cancer immunotherapy

**DOI:** 10.1002/EXP.20230115

**Published:** 2024-07-17

**Authors:** Qianqian Guo, Xiaoxuan Xu, Xiaojiang Lai, Jialin Duan, Dan Yan, Dangge Wang

**Affiliations:** ^1^ Precision Research Center for Refractory Diseases Shanghai General Hospital Shanghai Jiao Tong University School of Medicine Shanghai China; ^2^ State Key Laboratory of Drug Research & Center of Pharmaceutics Shanghai Institute of Materia Medica Chinese Academy of Sciences Shanghai China; ^3^ National Facility for Protein Science in Shanghai Shanghai Advanced Research Institute Chinese Academy of Sciences Shanghai China

**Keywords:** adjuvants, cancer vaccine, immunotherapy, liposome, surface charge

## Abstract

Cancer vaccines are promising to treat malignancy by delivering antigens and adjuvants to elicit host immunity. Beyond aluminum adjuvants, liposomes show efficient adjuvant effects through regulating the accumulation, internalization and release of payloads. However, it remains unknown that whether the liposome will perform intrinsic adjuvant effects in the absence of antigens and adjuvants. Herein, a library of antigen/adjuvant‐free liposomes with variable surface charges has been developed and it has been found that highly anionic liposomes show promising adjuvant effects for boosting immune responses. The anionic liposome mobilizes the MyD88 pathways of dendritic cells (DCs) to activate T helper cells and CD8^+^ T cells. The anionic liposomes enhance host immunity by regulating the population of Th1, Th2 and regulatory T cells (Tregs), and boost adaptive CD8^+^ T cells in lymphoid organs with good biosafety. It shows the most efficient protection against MC38 colorectal cancer in mice after a parallel injection of antigens and anionic liposomes. Overall, this study reveals that the surface charge of liposome affects its adjuvant efficiency and provides an anionic nanosized adjuvant formulation for enhancing immunization.

## Graphical abstract

In this study, the authors have developed a library of antigen/adjuvant‐free liposomes for testing the adjuvant effects. The anionic liposomes with appropriate surface charges enhance host immunity by regulating the Th1, Th2 and CD8^+^ T cells in lymphoid organs with good safety. It shows efficient therapy against MC38 colorectal cancer when combined with tumor antigens.

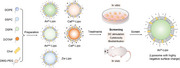

## 1 INTRODUCTION

Therapeutic cancer vaccines show great potential for treating advanced, metastatic and recurrent malignancies.^[^ ^1^ ^]^ It elicits host anti‐tumor immunity by initiating an immune cycle among tumor antigens, antigen‐presenting cells (APCs) and cytotoxicity T lymphocytes (CTLs).^[^ ^1^, ^2^ ^]^ For example, the mRNA vaccine encoding 34 tumor antigens (mRNA‐4157/V940) performs remarkable benefits against melanoma when combined with pembrolizumab.^[^ ^3^ ^]^ Adjuvants play a vital role in boosting the immunization effects of antigens.^[^ ^4^ ^]^ It facilitates the antigen‐presenting process by prolonging antigen retention, improving internalization, activating pattern recognition receptor (PRRs) and other undefined mechanisms.^[^ ^5^ ^]^ Although a diversity of vaccines has been commercialized, the approved adjuvants are quite limited, with only a few types such as aluminum salts, Toll‐like receptor (TLR) agonists and nano/micro‐sized drug carriers.^[^ ^5^, ^6^ ^]^ In most cases, the adjuvant is approved in pairs with specific antigens, lacking a universal feature and a clear clarification of its molecular mechanisms.^[^ ^7^ ^]^ Therefore, it shows a great priority to develop new adjuvants with broad application and clarify its adjuvant mechanisms in vivo.

Liposomes have been successfully used for delivering chemotherapeutics as well as mRNA in clinic.^[^ ^8^ ^]^ Several COVID‐2019 mRNA vaccines authorized by the USA Food and Drug Administration (FDA) are liposomal formulations.^[^ ^9^ ^]^ Conventionally, the liposome is considered to improve the transportation, internalization and release of antigens in APCs, especially in dendritic cells (DCs), so the antigens must be stably loaded and delivered.^[^ ^10^ ^]^ Besides, the surface charges and diameter of liposomes are optimized according to concerns about lymphoid targeting and stability, but this partly ignores the intrinsic adjuvant capability of the carriers.^[^ ^11^ ^]^ It has been reported that the surface charges of nanocarriers could mobilize immune responses.^[^ ^12^ ^]^ For instance, cationic drug carriers such as the cationic emulsion, polyethyleneimine (PEI) and ionizable polyamine show potent functions to enhance immune response.^[^ ^13^ ^]^ Similar adjuvant effects are found in cationic liposomes or lipid nanoparticles.^[^ ^14^ ^]^ However, the molecular mechanisms for surface charge‐induced immunity are mixed.^[^ ^15^ ^]^ It is struggling that the immune activation is attributed to the carrier‐promoted internalization or the surface charge itself.^[^ ^12^, ^14^ ^]^ It remains lacking a clear definition between the adjuvant effect of liposomes and its surface charge, especially for those with negative surface charges rather than cationic ones.^[^ ^10^, ^16^ ^]^ Given that cationic carriers may induce biosafety risks, the negatively charged liposomes show much promising potential in clinical translation if enabling adjuvant motivation.^[^ ^17^ ^]^ Besides, previous studies have revealed that higher level of positive ζ potential triggers higher toxicity in cells, suggesting the ζ potential levels may affect biological performance.^[^ ^18^ ^]^ But whether the degree of ζ potential will change the adjuvant effects of liposomes is unclear. Based on above concerns, exploring the correlation between liposomal surface charge and its adjuvant potency may facilitate the development of a universal nanosized adjuvant for enhancing cancer immunotherapy.

**SCHEME 1:**
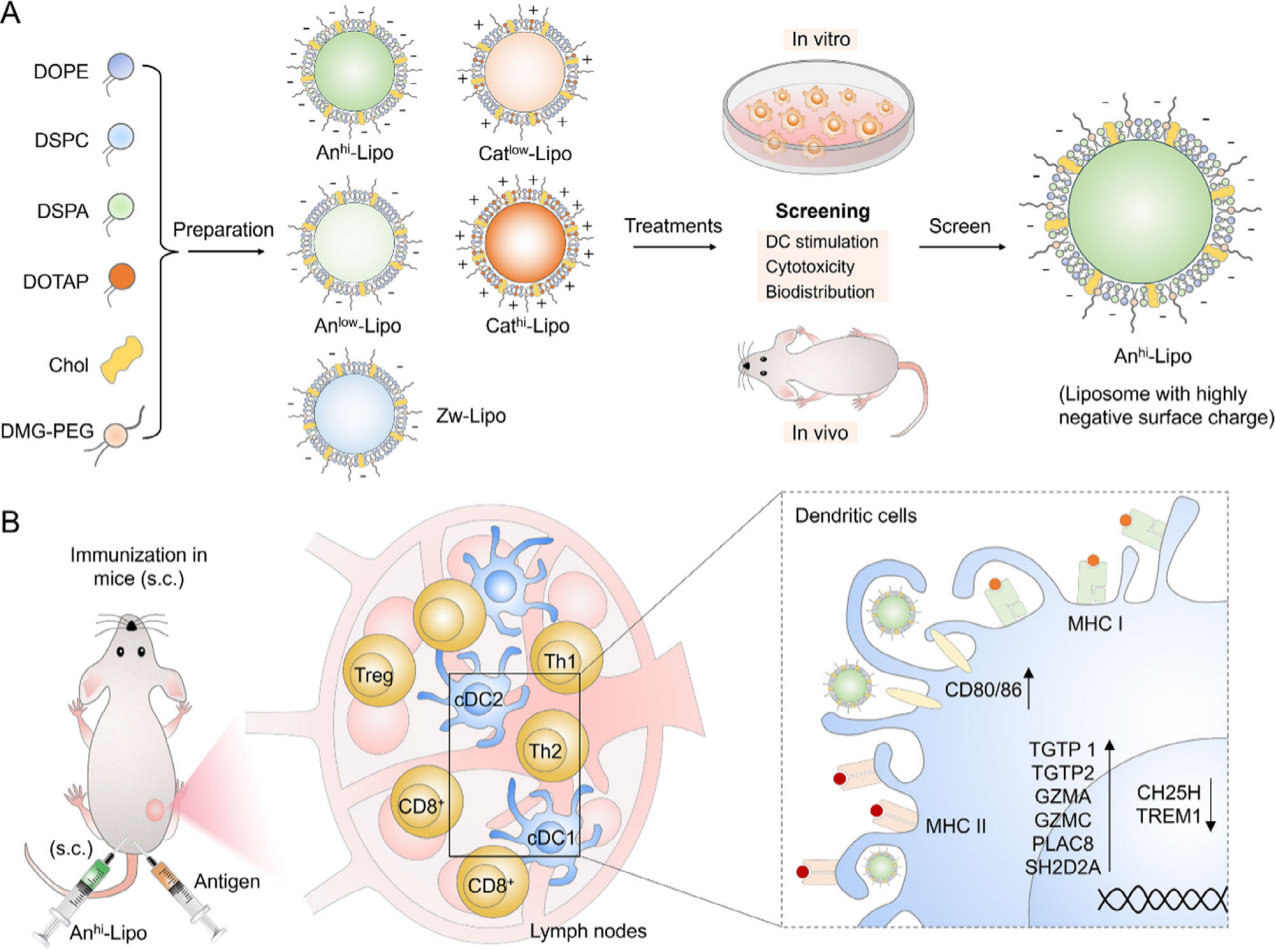
Scheme illustrating the development of charged liposomes and the adjuvant effects of anionic liposomes in vivo. (A) Fabrication of charged liposomes with different lipids and in vitro and in vivo screening process for adjuvant liposome. (B) The highly negative charged liposome shows effective adjuvant effects in vivo. It regulates the population of T helper cells and CD8+ T cells in lymph nodes. This could be attributed to the upregulation of TGTP1, TGTP2, GZMA, GZMC, PLAC8, SH2D2A and downregulation of CH25H and TREM1 in DCs post the treatment of anionic liposomes. The expression of CD80/86 was also improved after the treatments.

Herein, we have developed a library of antigen/adjuvant‐free liposomes with varied surface charges and screened five representative ones for exploring the correlation between surface charge and adjuvant effect (Scheme 1A). The surface charges of liposomes are adjusted via changing the composition of cationic, anionic and zwitterionic lipids. Then, the physicochemical features of liposomes are examined and its capabilities to improve the functions of DCs will be explored.^[^ ^19^ ^]^ In addition, RNA sequencing is performed to investigate the molecular changes in DCs after treated by charged liposomes.^[^ ^20^ ^]^ After that, the biodistribution of different charged liposomes is evaluated in mice and sequentially the immune responses are determined (Scheme 1B). Given that the surface charges may affect broad types of immune cells, the DCs, T helper cells and CD8^+^ T cells in immunized mice will be examined. Once confirming the adjuvant effects of certain liposomes, the anti‐tumor study will be performed in colorectal tumor‐bearing mouse models through parallelly treating tumor antigens and the antigen/adjuvant‐free liposomes. If successfully processed, this study will clarify the correlation between liposomal surface charge and its adjuvant effect, and provide a universal antigen/adjuvant‐free nanosized adjuvant.

## 2 RESULTS AND DISCUSSION

### 2.1 Preparation and characterization of liposomes with different surface charges

**FIGURE 1:**
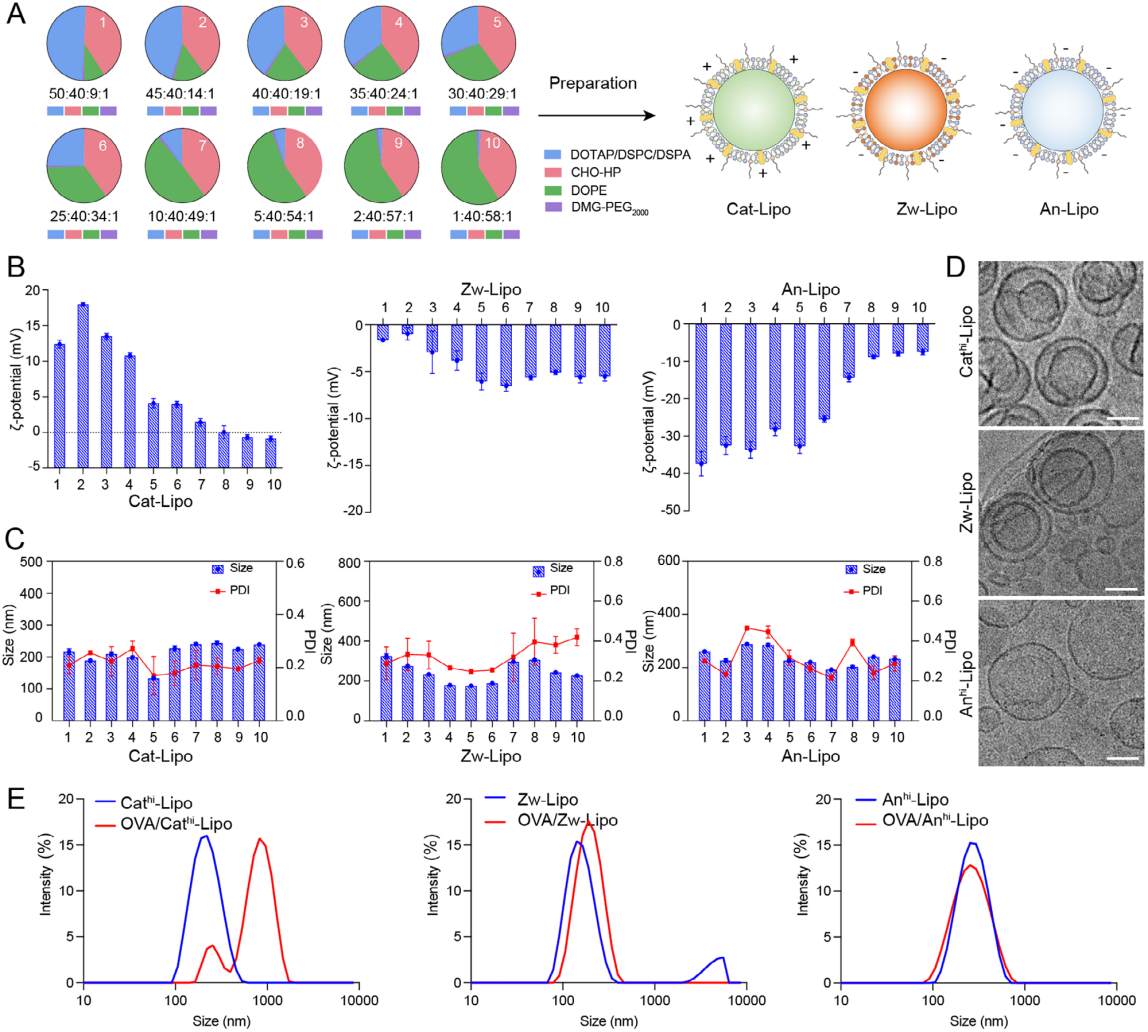
Fabrication and characterization of charged liposomes. (A) The liposomes were prepared by changing the ratio of cationic, anionic or zwitterionic lipids. (B) The ζ‐potential of anionic, zwitterionic and cationic liposomes of various lipid contents. The label of 1–10 is corresponding to the ratios in A. (C) The size of anionic, zwitterionic and cationic liposomes of various lipid contents. (D) Cryo‐TEM images of the CathiLipo, Zw‐Lipo and Anhi‐Lipo. Scale bars = 100 nm. (E) Size of Cathi‐Lipo, Zw‐Lipo and Anhi‐Lipo before and after mixed with OVA.

To prepare the liposomes with different surface charges, cholesterol (CHO‐HP), 1, 2‐dimyristoyl‐rac‐glycero‐3‐methoxypolyethylene glycol‐2000 (DMG‐PEG_2000_) and 1, 2‐dioleoyl‐sn‐glycero‐3‐phosphoethanolamine (DOPE) were selected as the basic compositions. Then, the surface charges of liposome were adjusted by changing the contents of the last composite lipid, cationic 1, 2‐dioleoyl‐3‐trimethylammonium‐propane (DOTAP), or zwitterionic 1, 2‐distearoyl‐sn‐glycero‐3‐phosphocholine (DSPC), or anionic lipid 1, 2‐distearoyl‐sn‐glycero‐3‐phosphate (DSPA). The molar ratio of ionizable lipid, CHO‐HP, DOPE and DMG‐PEG_2000_ was changed from 50:40:9:1 to 1:40:58:1. And the molar ratio between CHO‐HP and DMG‐PEG_2000_ was fixed at 40:1. As illustrated, a series of LNPs was prepared with cationic (Cat‐Lipo), zwitterionic (Zw‐Lipo) and anionic (An‐Lipo) lipid components and characterized by dynamic light scattering (DLS) and transmission electron microscopy (TEM), respectively (Figure 1A). The size, polymer dispersity index (PDI) and zeta potential of liposomes were examined and showed (Figure 1B,C). The surface charge of liposomes could be precisely regulated via changing the contents of ionizable lipids, ranging from −40 to 18 mV (Figure 1B). Then we selected the formulations including Cat^hi^‐Lipo (45% DOTAP), Zw‐Lipo (30% DSPC) and An^hi^‐Lipo (45% DSPA) as representative ones for examination. The morphology of these liposomes showed a double‐layer or multi‐layer spherical structure (Figure 1D), and the size was ≈130–293 nm (Figure 1C). Subsequently, we measured the stability of Cat^hi^‐Lipo (45% DOTAP), Zw‐Lipo (30% DSPC) and An^hi^‐Lipo (45% DSPA) at 37 and 4°C, respectively. The results showed that the size of these liposomes did not significantly change within 7 days (Figure S1A,B). To perform the diameter of liposomes in the presence of antigens, we mixed these liposomes with ovalbumin (OVA) proteins antigens, and measured the diameters before and after mixing by DLS. The results showed that the particle size and PDI increased significantly after mixing Cat^hi^‐Lipo with OVA. However, the diameter of An^hi^‐Lipo/OVA complex was not significantly changed after mixing (Figure 1E). This could be attributed to the electrostatic interactions between negatively charged OVA proteins and cationic liposomes.

### 2.2 Adjuvant effects of liposomes with varied surface charges in vitro

**FIGURE 2:**
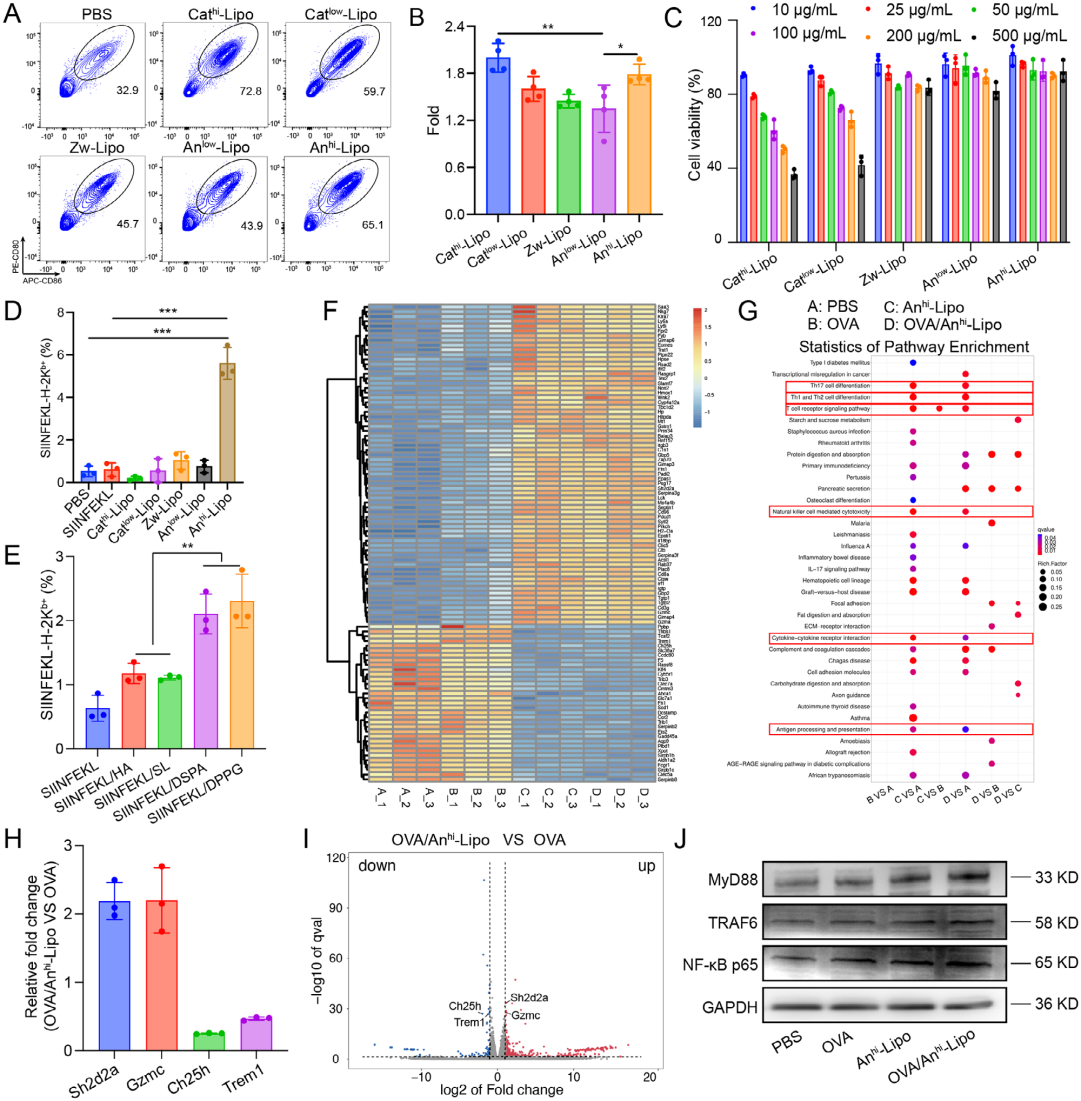
Adjuvant effects of charged liposomes in vitro. (A) BMDCs were incubated with different antigen/adjuvant‐free liposomes and the expression of CD80/CD86 was examined. (B) Increase fold of mature DCs after being treated by charged liposomes when compared to the PBS group. (C) Cell viability of BMDCs after incubated with liposomes at different concentrations (*n* = 3). (D) Presentation of SIINFEKL on BMDCs in control and treated groups. (E) Presentation of SIINFEKL on BMDCs in different groups. (F) Heatmap of top differentially expressed 100 genes in PBS (A_1‐3), OVA (B_1‐3), Anhi‐Lipo (C_1‐3) and OVA/Anhi‐Lipo (D_1‐3) groups. (G) Statistics of KEGG enrichment among four groups. (H) When compared to OVA group, OVA/Anhi‐Lipo induced upregulation of Sh2d2a and Gzmc while downregulation of Ch25h and Trem 1 in DCs. (I) Volcano maps showed the top differential genes between the compared two groups. (J) Expression of MyD88, TRAF6 and NF‐κB p65 with different treatments. *P < 0.05, **P < 0.01, ***P < 0.001.

Based on the ζ potential of liposomes, we screened five kinds of liposome with varied surface charges, including Cat^hi^‐Lipo (45% DOTAP), Cat^low^‐Lipo (2% DOTAP), Zw‐Lipo (30% DSPC), An^low^‐Lipo (2% DSPA), and An^hi^‐Lipo (45% DSPA). Bone marrow‐derived DCs (BMDCs) from C56BL/6 mice were used to evaluate the adjuvant effects of charged liposomes. The immature BMDCs were incubated with the five kinds of liposomes, respectively. Then, the co‐stimulatory receptor CD80/CD86 on BMDCs were examined (Figure S2). It was found that Cat^hi^‐Lipo and An^hi^‐Lipo performed the most efficient capability to enhance the expression of CD80/CD86 (Figure 2A,B). The results show that An^hi^‐Lipo indeed promoted the maturation of BMDCs. Then we determined the cell viability of BMDCs after incubating with the charged liposomes. Consistently, cationic liposomes including Cat^hi^‐Lipo and Cat^low^‐Lipo showed obvious cytotoxicity in BMDCs. The viability of BMDCs was sharply decreased even with 200 µg mL^−1^ of cationic liposomes (Figure 2C). The anionic liposomes did not show obvious cytotoxicity even at 500 µg mL^−1^. Given to the cell viability, the An^hi^‐Lipo showed much promising potential as a nanosized adjuvant. To further clarify the ideal potential of anionic liposomes as an adjuvant, we prepared eight formulations with electronegative intensity, with the proportion of DSPA ranging from 55% to 20%, respectively (Supplementary Table 1). Through the measurement of size and ζ potential, the results showed that the surface potential range was from −19.9 to −40.30 mV (Figure S3), which was basically consistent with the results in Figure 1B,C. Furthermore, we investigated the adjuvant effect of this series of liposomes. The results showed that the adjuvant effects were significant when the surface charges of liposomes ranged between −27.8 and −40.30 mV. In addition, we also compared the effects of An^hi^‐Lipo and MF59 for inducing mature BMDCs in vitro. BMDCs were incubated with An^hi^‐Lipo or commercially available adjuvant MF59 for 24 h, respectively. PBS‐treated group was set as a negative control group. The results showed that the An^hi^‐Lipo showed much similar adjuvant effects for promoting DCs maturation when compared to the MF59 (Figure S4). Subsequently, we verified the adjuvant effects of An^hi^‐Lipo with OVA antigens. The presentation of OVA_257‐264_ (SIINFEKL) peptides was determined by flow cytometry (Figure S5).^[^ ^21^ ^]^ Highest level of antigen presentation was found in An^hi^‐Lipo group compared to other liposome groups (Figure 2D), indicating that An^hi^‐Lipo presented the most significant presentation effect when combined with antigens. Furthermore, lipids with sulfonic acid group (sodium laurylsulfonate, SL), carboxylic acid group (2‐hexadecyloctadecanoic acid, HA) and phosphate groups (1,2‐distearoyl‐sn‐glycero‐3‐phosphat, DSPA and 1,2‐dipalmitoyl‐sn‐glycero‐3‐PG sodium, DPPG) were used to prepare the liposomes with similar surface charges. BMDCs were incubated with the corresponding liposomes at equal concentrations. The results showed that the proportion of mature DCs in DSPA and DPPG groups was significantly increased, while that in HA or SL group was not elevated (Figure S6). Accordingly, when combined with antigen peptide SIINFEKL, DSPA and DPPG significantly enhanced the presentation of SIINFEKL in DCs (Figure 2E). These results indicated that the adjuvant effect of anionic lipids might be derived from the phospholipid groups.

To further investigate the adjuvant mechanisms of anionic liposome An^hi^‐Lipo, RNA sequencing was performed on BMDCs treated with PBS, OVA, An^hi^‐Lipo and OVA/An^hi^‐Lipo, respectively (Figure S7). As shown (Figure 2F), the expression level of more than 100 genes was significantly changed. Through Pearson correlation coefficient and principal component analysis of gene samples from different groups, the samples showed good repeatability and could be used for subsequent analysis (Figure S8). The GO enrichment and KEGG enrichment analysis was performed for the differential genes among the groups to define the altered signaling pathways. In GO enrichment, the terms related to T cell receptor complex, inflammatory response, immune system process, immune response and adaptive immune response were obviously changed after treated by An^hi^‐Lipo (Figure S9). In KEGG enrichment, obvious changed pathways related to Th1, Th2 and Th17 cell differentiation, T cell receptor signaling pathway and antigen processing/presentation were detected in An^hi^‐Lipo group (Figure 2G). The changes of representative RNA were shown. Among these key genes, the expression of Tgtp1, Tgtp2, Gzma, Gzmc, Plac8, Sh2d2a increased significantly, while Ch25h and Trem1 decreased in the presence of An^hi^‐Lipo (Figures 2H and S10). These genes are closely related to the presentation of exogenous antigens, metabolism of lipids and the proliferation of DCs. A volcano map of differentially expressed genes between the two groups was performed (Figures 2I and S10). Based on the enrichment, it was found that key pathways related to major histocompatibility complex (MHC) I and MHC II expression were changed, thus would affect the regulation of CD4^+^ T cell and the cross‐presentation to CD8^+^ T cells. Besides, we extracted total proteins of BMDCs treated with PBS, OVA, An^hi^‐Lipo and OVA/An^hi^‐Lipo, and evaluated the expression of key proteins in the pathway of TLR agonists, such as MyD88, TRAF6 and NF‐κB p65. As shown, the results confirmed that the expression levels of MyD88, TRAF6 and NF‐κB p65 were elevated in the An^hi^‐Lipo group (Figure 2J), indicating that the adjuvant effect of An^hi^‐Lipo was similar to that of the commonly used TLR agonists. The An^hi^‐Lipo could not only provide adjuvant effects without loading antigens, but also serve as delivery vehicles for payloads. According to the results, An^hi^‐Lipo boosted the DCs via the MyD88‐TRAF6 pathway, the An^hi^‐Lipo may enable a broad application in vaccines with its intrinsic adjuvant effects.

### 2.3 In vivo distribution of liposomes with varied surface charges

**FIGURE 3:**
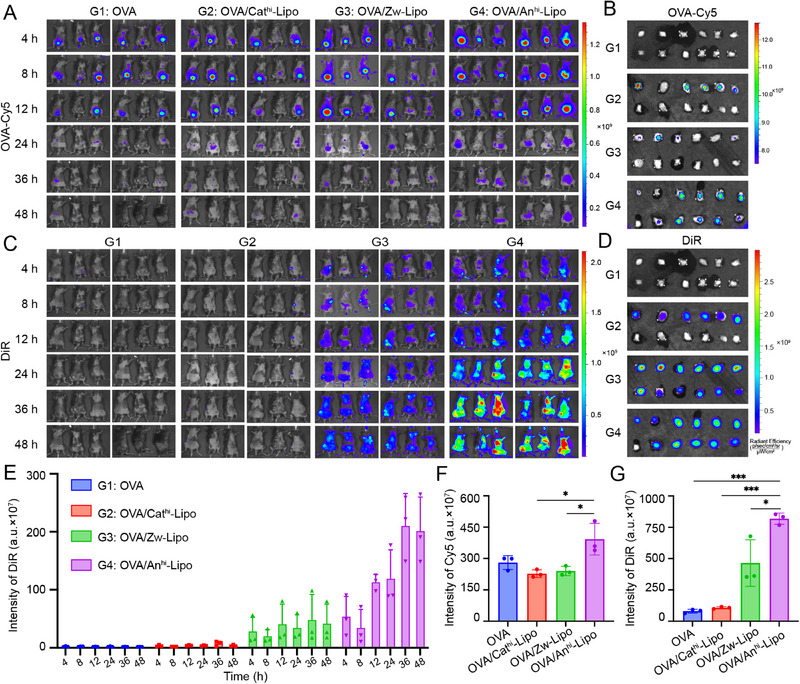
Biodistribution of OVA‐Cy5 and DiR‐labeled liposomes in vivo. OVA‐Cy5 and DiR‐labeled liposomes were parallelly treated via subcutaneous injection. Then the fluorescent signals of OVA‐Cy5 and DiR were examined from two channels. G1, OVA; G2, OVA/Cathi‐Lipo; G3, OVA/Zw‐Lipo; G4, OVA/AnhiLipo. (A) The distribution of OVA‐Cy5 in mice at different time points. (B) Examining the fluorescence signals of OVA‐Cy5 in lymph nodes ex vivo. (C) The distribution of DiR in mice. (D) Fluorescent signals of DiR in lymph nodes ex vivo. (E) Quantification of DiR signal intensity in mice. (F) f were isolated from the lymph nodes at 48 h and examined by flow cytometry for Cy5+ DCs, and (G) DiR+ DCs. *P < 0.05, ***P < 0.001.

Inspired by the potential adjuvant effects of negatively charged liposome in vitro, we evaluated the in vivo distribution for further exploration. Fluorescence dye DiR was used to label the liposomes, and parallelly subcutaneously administrated with OVA‐Cy5 for imaging. According to the results, OVA‐Cy5 mainly distributed in the hypogastria of mice, and the retention of OVA‐Cy5 was slightly prolonged when combined with An^hi^‐Lipo (Figure 3A). This feature could be clearly observed in the ex vivo images of lymph nodes after treated for 48 h (Figure 3B). Since the OVA‐Cy5 was not loaded by the liposomes, the distribution of DiR‐labeled liposomes was also performed. Obvious signals were detected across the belly of mice in An^hi^‐Lipo and Zw‐Lipo groups (Figure 3C). Besides, the accumulation of anionic liposomes in lymph nodes was higher than that treated by cationic liposomes (Figure 3D).

The quantification of fluorescence intensity also showed that anionic liposomes preferred to drain and accumulate in lymph nodes (Figures 3E and S11). This behavior was also proven in our previous studies.^[^ ^22^ ^]^ After the imaging, lymph nodes were collected and the proportion of Cy5 and DiR‐positive cells in the lymph nodes was analyzed by flow cytometry. The results showed that the proportion of Cy5 and DiR‐positive DCs in OVA/ An^hi^‐Lipo group was significantly higher than that in other groups (Figure 3F,G). The above results confirmed that negatively charged liposomes were efficiently distributed and accumulated in lymph nodes.

### 2.4 Anionic liposomes regulated T helper cells and CD8^+^ T cells in vivo

**FIGURE 4:**
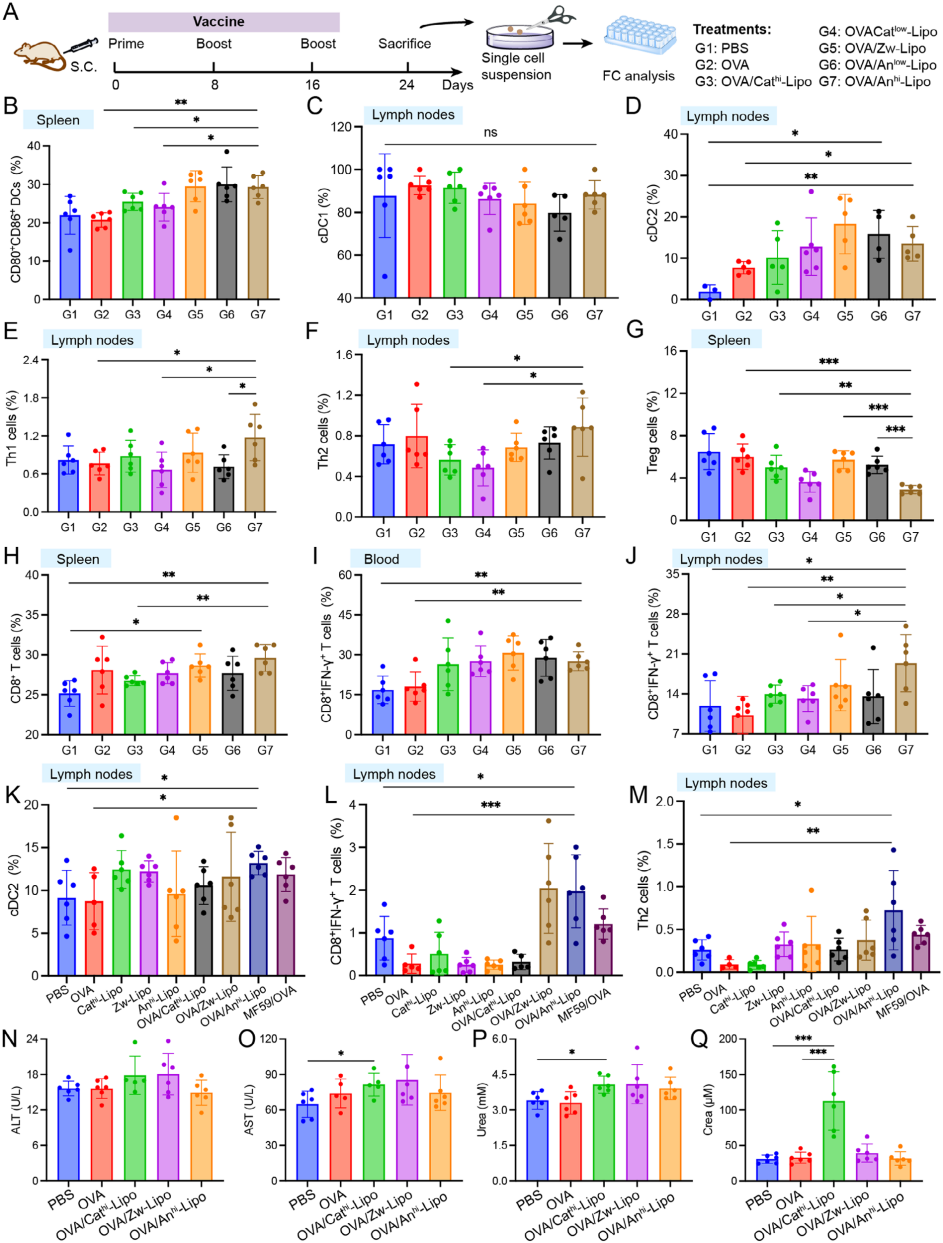
Charged liposomes induced immune responses in vivo. (A) Schematic illustration of prime and boost timeline and immune analysis in vivo. (B) Population of mature DCs in spleen. (C) Population of cDC1 (gated on CD45+CD11c+Lin‐F4/80‐), and (D) cDC2 (gated on CD45+CD11c+Lin‐F4/80‐) in lymph nodes in control and treated groups (n = 4‐6). (E) Frequency of Th1 cells (gated on CD4+ T), and (F) Th2 cells (gated on CD4+ T) in lymph nodes after different treatments. (G) Population of Tregs (gated on CD4+ T) in spleen. (H) Population of CD8+ T cells in spleen. (I) Frequency of CD8+IFN‐γ+ T cells in blood, and (J) lymph nodes in control and treated groups. (K) cDC2 (gated on CD45+CD11c+ Lin‐ F4/80‐) in lymph nodes in control and treated groups. (L) Frequency of CD8+ IFN‐γ+ T cells in lymph nodes. (M) Th2 cells (gated on CD4+ T) in lymph nodes after different treatments. (N) Serum level of ALT, (O) AST, (P) urea, and (Q) crea after various treatments (*n* = 6). *P < 0.05, **P < 0.01, ***P < 0.001.

To evaluate the adjuvant effects of charged liposomes, analysis of the immune responses was performed in BALB/c mice. Briefly, the mice were parallelly treated with OVA antigens and liposome suspensions, and then examined at desired time points. The peripheral blood, spleen and lymph node samples were collected one week after the secondary administration (Figure 4A). Firstly, the maturation of DCs was examined after the treatments (Figures S12 and S13). It was found that the anionic liposomes promoted the maturation of DCs in vivo (Figure 4B). Besides, the population of classical type 1 DCs (cDC1) and cDC2 was determined. It has been confirmed that the cDC1 is responsible for adaptive T cell immunity, especially for the cross‐presentation of CD8^+^ T cells, while the cDC2 shows a higher association with the regulation of T helper cells such as Th1, Th2 and Th17 cells.^[^ ^23^ ^]^ The results showed that there was no significant change in the population of cDC1 in lymph nodes, but the negatively charged liposomes significantly enhanced the population of cDC2 (Figure 4C,D). Interestingly, the OVA/Cat^hi^‐Lipo did not improve cDC2 in lymph nodes, which might be due to the fact that the aggregate of anionic OVA and cationic liposomes suppressed its drainage to lymph nodes. Based on the enhanced cDC2 population and RNA‐sequencing results, we next explored the frequency of Th1, Th2 and Th17 cells in lymph nodes. The results showed that OVA/An^hi^‐Lipo enhanced the frequency of Th1 cells when compared to free OVA or OVA/An^low^‐Lipo (Figure 4E). Compared with OVA/Cat^hi^‐Lipo and OVA/Cat^low^‐Lipo, higher population of Th2 cells was detected in OVA/An^hi^‐Lipo (Figure 4F). Neither the cationic nor the anionic liposomes affected the population of Th17 cells (Figure S14). Since the Tregs play a suppressive role in immune activation, its population was also determined. The OVA/An^hi^‐Lipo reduced the population of Tregs in spleen, showing a much higher efficiency than OVA/An^low^‐Lipo (Figure 4G). Accordingly, a higher negative surface charge may help with the regulation of Th1 and Tregs in vivo. Furthermore, the population of CD8^+^ T cells was evaluated. Zw‐Lipo and An^hi^‐Lipo enhanced the population of CD8^+^ T cell in spleen (Figure 4H). The level of IFN‐γ^+^CD8^+^ T cell in blood and lymph node (Figure 4I,J) was also enhanced in OVA/An^hi^‐Lipo group. Then we constructed MC38‐OVA tumor‐bearing mice model for evaluating the immunity. The mice were divided into nine groups (PBA, OVA, Cat^hi^‐Lipo, Zw‐Lipo, An^hi^‐Lipo, OVA/Cat^hi^‐Lipo, OVA/Zw‐Lipo, OVA/An^hi^‐Lipo and MF59/OVA). The mice in each group were treated with the vaccine for three times every 5 days, and then inoculated with MC38‐OVA subcutaneous tumors. The spleen and lymph nodes of mice in each group were collected 20 days later, and the results were consistent with the performance of the immune responses in BALB/c mice. Negatively charged liposomes significantly enhanced the population of mature DCs and cDC2 (Figures 4K and S15). Besides, the results showed that OVA/An^hi^‐Lipo enhanced the frequency of Th2 cells when compared to free OVA or PBS, as well as MF59/OVA. Furthermore, the population of IFN‐γ^+^ CD8^+^ T cells were evaluated. The level of IFN‐γ^+^CD8^+^ T cells in lymph node and spleen was also enhanced in OVA/An^hi^‐Lipo group (Figures 4L,M and S15). These results suggested that the anionic liposome with highly negative charges could mobilize a systemic immune response to combat tumors. At the same time, to evaluate the biosafety of charged liposomes, the plasma was collected and examined to determine liver function indicators (alanine transaminase [ALT, Figure 4N], aspartate aminotransferase [AST, Figure 4O]) and renal function indicators (urea [Figure 4P] and crea [Figure 4Q]). Anionic liposomes did not induce alternation of these molecules, while high level of AST, urea and crea was detected in Cat^hi^‐Lipo‐treated mice. These results suggested that cationic liposome might trigger biosafety risks in vivo. In addition, major organs including heart, liver, spleen, lung and kidney were collected for hematoxylin and eosin (H&E) staining. Pathological injuries were found in liver, spleen and lung of BALB/c and C57BL/6 mice with Cat^hi^‐Lipo treatment (Figures S16 and S17). These results indicate that An^hi^‐Lipo is promising to serve as a nanosized adjuvant with good biosafety.

### 2.5 Anti‐tumor study in MC38‐OVA mouse tumor model

**FIGURE 5:**
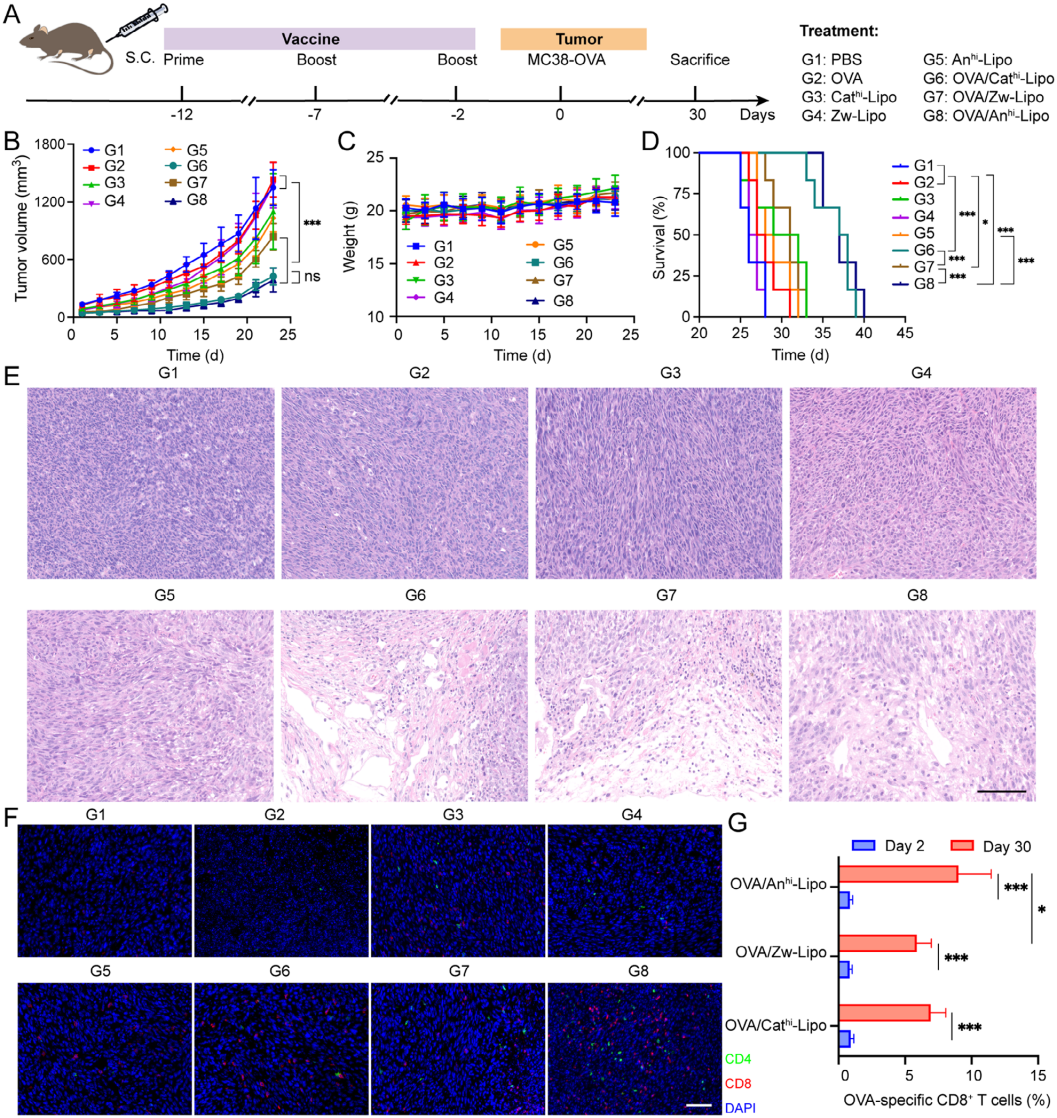
Anti‐tumor study in MC38‐OVA mouse model. (A) Schematic illustration of the treatment project in MC38‐OVA prophylactic tumor model. (B) Growth kinetic of tumors after various treatments (*n* = 6). (C) Body weight of mice during the study. (D) Survival of mice in control and treated groups. (E) H&E staining of tumor sections at the end of the study. Scale bars = 200 µm. (F) Fluorescent staining of CD4+ (green) and CD8+ (red) T cells in tumors. Scale bars = 200 µm. (G) Proportion of OVA‐epitope specific CD8+ T cells in blood of mice on day 2 and day 30 post the MC38‐OVA inoculation. *P < 0.05, **P < 0.01, ***P < 0.001.

Given to the immune activation induced by anionic liposomes, we next investigated the anti‐tumor effects in a colorectal tumor model. The C57BL/6 mice were vaccinated with parallelly treated OVA proteins antigens and liposomal suspensions for three times. It would prime and boost the anti‐tumor immune responses in mice. After that, MC38‐OVA tumor cells were inoculated in the vaccinated mice and the growth of tumors was monitored (Figure 5A). Based on the growth kinetics of MC38‐OVA tumors, OVA/Cat^hi^‐Lipo, OVA/Zw‐Lipo and OVA/An^hi^‐Lipo inhibited the growth of tumors. The tumor volume reached 1349.29 mm^3^ with PBS treatment on day 23, while that was only 387.54 mm^3^ when treated by OVA/An^hi^‐Lipo (Figures 5B and S18). No significant decrease of mouse body weight was detected in all groups (Figure 5C). Both the OVA/Cat^hi^‐Lipo and OVA/An^hi^‐Lipo prolonged the survival of MC38‐OVA tumor‐bearing mice (Figure 5D). The mice treated by PBS were died within 30 days. Antigen/adjuvant‐free liposomes could slightly prolong the survival of mice. Mice treated by OVA/Cat^hi^‐Lipo and OVA/An^hi^‐Lipo showed significantly extended lifetime. Subsequently, H&E and immunofluorescence staining were performed on the tumors of each group. Obvious necrosis was observed in the tumor sections from OVA/Cat^hi^‐Lipo, OVA/Zw‐Lipo and OVA/An^hi^‐Lipo groups (Figure 5E). Meanwhile, the infiltration of CD4^+^ (green signal) and CD8^+^ T (red signal) cells was obviously stained in tumor sections from OVA/An^hi^‐Lipo group, indicating that anionic liposome could enhance the anti‐tumor T cell immunity in vivo (Figure 5F). Furthermore, we collected peripheral blood of mice on days 2 and 30, respectively, for detecting the OVA epitope‐specific CD8^+^ T cells (Figure S19). OVA‐epitope specific CD8^+^ T cells in OVA/Cat^hi^‐Lipo, OVA/Zw‐Lipo and OVA/An^hi^‐Lipo groups were increased from day 2 to day 30. Most high population of OVA epitope‐specific CD8^+^ T cells was achieved in OVA/An^hi^‐Lipo group (Figure 5G). The An^hi^‐Lipo could be a better choice for eliciting immunity in company with the antigens for enhancing cancer immunotherapy.

## 3 CONCLUSION

In this manuscript, we have developed an anionic antigen/adjuvant‐free liposome which shows highly efficient adjuvant effect for enhancing cancer immunotherapy. The anionic liposome improves the maturation of DCs and facilitates antigen presentation process. The results show that the anionic liposome mobilizes immune responses through activating MyD88‐TRAF6 pathways in DCs. For in vivo distribution, anionic liposomes show most efficient efficiency to accumulate in lymph nodes, which is benefit from the negative surface charge. The An^hi^‐Lipo performs a positive regulation on Th1 cells and enables higher population of Th2 cells in contrast to cationic liposomes. Besides, it also mobilizes the adaptive T cell immunity and enhanced the population of CD8^+^ T cells. When combined with OVA antigens, the OVA/An^hi^‐Lipo could improve the inhibition against MC38‐OVA colorectal cancer and prolong the survival of mice. The most important finding of this study is that highly anionic liposome also maintains adjuvant effects for enhancing immune responses. Conventionally, cationic polymers and carriers are confirmed with immunity‐stimuli capability but the negatively charged carriers are ignored. However, in practice the cationic ones face obvious biosafety challenges which limits its application. The highly anionic liposomes not only enable adjuvant effects, but also show good biosafety according to the cytotoxicity evaluation and IHC examination of major organs. This could be the most promising potency for clinical translation of the nanosized adjuvants. Overall, this study has clarified the adjuvant potency of highly anionic antigen/adjuvant‐free liposomes, and explore its mechanisms to mobilize DCs, which may provide an alternative strategy for developing nanosized adjuvant for improving cancer immunotherapy.

## 4 EXPERIMENTAL SECTION

Experimental details are provided in the Supporting Information.

## CONFLICT OF INTEREST STATEMENT

The authors declare no conflicts of interest.

## DATA AVAILABLILITY STATEMENT

All the data associated with this study are presented in the paper or in the Supporting Information.

## Supporting information

Supporting Information

## References

[1] M. J. Lin , J. Svensson‐Arvelund , G. S. Lubitz , A. Marabelle , I. Melero , B. D. Brown , J. D. Brody Nat. Cancer 2022, 3, 911. 35999309 10.1038/s43018-022-00418-6

[2] F. Lang , B. Schrörs , M. Löwer , Ö. Türeci , U. Sahin , Nat Rev. Drug Discovery 2022, 21, 261. 35105974 10.1038/s41573-021-00387-y PMC7612664

[3] A. Khattak , J. S. Weber , T. Meniawy , M. H. Taylor , G. Ansstas , K. B. Kim , M. McKean , G. V. Long , R. J. Sullivan , M. B. Faries , T. Tran , C. L. Cowey , T. M. Medina , J. M. Segar , V. Atkinson , G. T. Gibney , J. J. Luke , E. I. Buchbinder , R. S. Meehan , M. S. Carlino , b. M. A. Grp , J. Clin. Oncol. 2023, 41, LBA9503.

[4] W. J. Lee , M. Suresh , Front. Immunol. 2022, 13, 940047. 35979365 10.3389/fimmu.2022.940047 PMC9376467

[5] B. Pulendran , P. S. Arunachalam , D. T. O'Hagan , Nat. Rev. Drug Discovery 2021, 20, 454. 33824489 10.1038/s41573-021-00163-y PMC8023785

[6] T. Zhao , Y. Cai , Y. Jiang , X. He , Y. Wei , Y. Yu , X. Tian , Signal Transduction Targeted Ther. 2023, 8, 283. 10.1038/s41392-023-01557-7 PMC10356842 37468460

[7] a) A. M. Vargason , A. C. Anselmo , S. Mitragotri , Nat. Biomed. Eng. 2021, 5, 951; 33795852 10.1038/s41551-021-00698-w

[8] A. Mukherjee , B. Bisht , S. Dutta , M. K. Paul , Acta Pharmacol. Sin. 2022, 43, 2759. 35379933 10.1038/s41401-022-00902-w PMC9622806

[9] K. L. Swingle , A. G. Hamilton , M. J. Mitchell , Trends Mol. Med. 2021, 27, 616. 33836968 10.1016/j.molmed.2021.03.003

[10] N. Wang , M. Chen , T. Wang , J. Controlled Release 2019, 303, 130. 10.1016/j.jconrel.2019.04.025 PMC7111479 31022431

[11] a) Y. Xia , S. Fu , Q. Ma , Y. Liu , N. Zhang , Nano‐Micro Lett. 2023, 15, 145; 10.1007/s40820-023-01125-2 PMC10239433 37269391

[12] G. Zhu , Y.‐G. Yang , T. Sun , Biomater. Sci. 2022, 10, 1408. 35137771 10.1039/d2bm00011c

[13] a) A. W. Li , M. C. Sobral , S. Badrinath , Y. Choi , A. Graveline , A. G. Stafford , J. C. Weaver , M. O. Dellacherie , T. Y. Shih , O. A. Ali , J. Kim , K. W. Wucherpfennig , D. J. Mooney , Nat. Mater. 2018, 17, 528; 29507416 10.1038/s41563-018-0028-2 PMC5970019

[14] a) R. Zhang , L. Tang , Y. Tian , X. Ji , Q. Hu , B. Zhou , Z. Ding , H. Xu , L. Yang , J. Controlled Release 2020, 328, 210; 10.1016/j.jconrel.2020.08.023 32860927

[15] M. M. T. van Leent , B. Priem , D. P. Schrijver , A. de Dreu , S. R. J. Hofstraat , R. Zwolsman , T. J. Beldman , M. G. Netea , W. J. M. Mulder , Nat. Rev. Mater. 2022, 7, 465.

[16] M. Z. Ahmad , J. Ahmad , M. Y. Alasmary , B. A. Abdel‐Wahab , M. H. Warsi , A. Haque , P. Chaubey , Immunotherapy 2021, 13, 491. 33626936 10.2217/imt-2020-0258

[17] S. Zhou , Y. Luo , J. F. Lovell , Expert Rev. Vaccines 2023, 22, 1022. 37878481 10.1080/14760584.2023.2274479 PMC10872528

[18] a) Y. Zhang , N. V. Hudson‐Smith , S. D. Frand , M. S. Cahill , L. S. Davis , Z. V. Feng , C. L. Haynes , R. J. Hamers , J. Am. Chem. Soc. 2020, 142, 10814; 32402194 10.1021/jacs.0c02737

[19] R. Yu , Y. Mai , Y. Zhao , Y. Hou , Y. Liu , J. Yang , J. Drug Target 2019, 27, 780. 30589361 10.1080/1061186X.2018.1547734

[20] B. Chen , L. Zhu , S. Yang , W. Su , Front. Immunol. 2021, 12, 711329. 34566965 10.3389/fimmu.2021.711329 PMC8458576

[21] P. Xiao , J. Wang , Z. Zhao , X. Liu , X. Sun , D. Wang , Y. Li , Nano Lett. 2021, 21, 2094. 33622034 10.1021/acs.nanolett.0c04783

[22] L. Zhou , W. Yi , Z. Zhang , X. Shan , Z. Zhao , X. Sun , J. Wang , H. Wang , H. Jiang , M. Zheng , D. Wang , Y. Li , Natl. Sci. Rev. 2023, 10, nwad214. 37693123 10.1093/nsr/nwad214 PMC10484175

[23] D. A. Anderson , C. A. Dutertre , F. Ginhoux , K. M. Murphy , Nat. Rev. Immunol. 2021, 21, 101. 32908299 10.1038/s41577-020-00413-x PMC10955724

