# Supplementary material for: Antigen/adjuvant‐free liposome induces adjuvant effects for enhancing cancer immunotherapy
Source: Exploration (Beijing). 2024 Jul 17;5(2):20230115. doi: 10.1002/EXP.20230115 (PMC12087400; doi:10.1002/EXP.20230115)
Supplement: Supplementary file 1 — Supporting Information [file EXP2-5-20230115-s001.docx]

**Supporting information for**

**Antigen/adjuvant-free liposome induces adjuvant effects for enhancing cancer immunotherapy**

Qianqian Guo^1, 4^, Xiaoxuan Xu^2, 4^, Xiaojiang Lai^1^, Jialin Duan^3^, Dan Yan^2^, Dangge Wang^1,^ *

^1^Precision Research Center for Refractory Diseases, Shanghai General Hospital, Shanghai Jiao Tong University School of Medicine, Shanghai, 201620, China;

^2^State Key Laboratory of Drug Research & Center of Pharmaceutics, Shanghai Institute of Materia Medica, Chinese Academy of Sciences, Shanghai 201203, China;

^3^National Facility for Protein Science in Shanghai, Shanghai Advanced Research Institute, Chinese Academy of Sciences, Shanghai 201210, China.

^4^These authors contributed equally: Qianqian Guo, Xiaoxuan Xu.

[*] Corresponding authors: Prof. Dangge Wang (dg_wang@sjtu.edu.cn).

**Materials and methods**

**Materials**

1,2-Dioleoyl-3-trimethylammonium-propane (DOTAP), 1,2-distearoyl-sn-glycero-3-phosphocholine (DSPC), 1,2-Distearoyl -sn-glycero-3-phosphate (DSPA), Cholesterol (for injection) (CHO-HP), 1,2-Dioleoyl-sn-glycero-3-phosphoethanolamine (DOPE), 1,2-dimyristoyl-rac-glycero-3-methoxypolyethylene glycol-2000 (DMG-PEG_2000_) and MF59 (C01001) were purchased from AVT (shanghai) Pharmaceutical Tech Co., Ltd (Shanghai, China). OVA_257–264_ (SIINFEKL) peptide were purchased from BankPeptide biological technology Inc (Hefei, An’hui, China). VeZol Reagent (R411) was purchased from Vazyme Biotech Co., Ltd (Nanjing, Jiangsu, China). T-Select H-2Kb OVA Tetramer-SIINFEKL-PE (TS-5001-1C) was purchased from MBL Beijing biotech CO., LTD (Beijing, China). Zombie UV™ Fixable Viability Kit (423107), Zombie Aqua™ Fixable Viability Kit (423101), Brilliant Violet 510™ anti-mouse CD45 (103138), PerCP/Cyanine5.5 anti-mouse CD11c (117327), PE anti-mouse CD80 (104707), Brilliant Violet 785™ anti-mouse CD86 (105043), APC anti-mouse CD86 (105011), FITC anti-mouse CD3 (100204), FITC anti-mouse CD19 (101505), FITC anti-mouse CD49b (103503), FITC anti-mouse Ly-6G (127605), Brilliant Violet 605™ anti-mouse/rat XCR1 Antibody (148222), PE/Cyanine7 anti-mouse CD172a (SIRPα) (144008), Brilliant Violet 650™ anti-mouse F4/80 (123149), Brilliant Violet 650™ anti-mouse CD4 (100555), PE/Cyanine7 anti-mouse CD8a (100722), Brilliant Violet 605™ anti-mouse IFN-γ (505840), Brilliant Violet 421™ anti-mouse IL-4 (504127), Brilliant Violet 510™ anti-mouse IL-17A (506933), and PE anti-mouse/rat/human FOXP3 (320008) were purchased from Biolegend (San Diego, California, USA). OVA protein (A5503) was purchased from Sigma (St. Louis, USA). DiR, Cy5, CCK-8 kit, RPMI 1640 medium, penicillin/streptomycin solution, fetal bovine serum (FBS) and red blood cell lysis buffer were purchased from meilunbio (Dalian, Liaoning, China). Recombinant murine GM-CSF (315-03) and recombinant murine IL-4 (214-14) were purchased from Pepro Tech (New Jersey, USA). MC38-OVA cells were purchased from the cell bank of the Chinese Academy of Sciences (Shanghai, China). C57BL/6 and BALB/c mice were purchased from Vital River (Beijing, China). All animal experiments were conducted in accordance with the experimental protocol approved by the Experimental Animal Management Committee of Shanghai General Hospital (2023AW039).

**Preparation and characterization of charged liposomes**

DOTAP, DSPC and DSPA were dissolved in trichloromethane or a mixture of trichloromethane and methanol respectively, and ultrasonic cleaning instrument was used for 10 s to promote their full dissolution. Then CHO-HP, DOPE and DMG-PEG_2000_ were dissolved in trichloromethane, and all the above materials were transferred to a round-bottomed flask. Molar ratio of DSPA (or DOTAP, DSPC), CHO-HP, DOPE and DMG-PEG_2000_ was 50:40:9:1~ 1:40:58:1 (molar ratio). After a water bath at 60 ℃ for a few seconds, the lipids were dried in a water bath at 37 ℃. After 4-5 min, the lipids could be seen as a film attached to the round-bottom flask wall. 2 mL of PBS was added to the round-bottom flask, and then stirred in a water bath at 60 ℃ for 2 h, fully hydrated, and then homogenized by ultrasound, respectively. The different charged liposomes were prepared with the same methods. Size distribution and zeta potential of Cat-Lipo, Zw-Lipo and An-Lipo were detected by DLS examination (ZS90, Malvern). The morphology of liposomes was captured by transmission electron microscopy (TEM) (Talos L120C, FEI).

**Maturation and antigen-presentation of BMDCs in vitro**

Bone marrow monocytes were collected from 8-week C57BL/6 male mice. The cell concentration was adjusted to 10^6^ cells mL^-1^, and the cytokine GM-CSF (20 ng mL^-1^) and IL-4 (10 ng mL^-1^) were supplemented. Cell suspensions were inoculated into 6-well plates. Two days later, the medium in plate was removed, fresh 1640 medium was supplemented, and the cytokines were supplemented. Three days later, mouse bone marrow derived dendritic cells (BMDC) was obtained. For cytotoxicity assay, the BMDCs obtained by the above method were inoculated into a 96-well cell culture plate with 5×10^5^ cells well^-1^. Then BMDCs was incubated with liposomes for 48 h at different concentrations (10, 25, 50, 100, 200 or 500 μg mL^-1^). The cells without treatment and with PBS were set as control groups. CCK-8 kit was added to the cell culture plate for examining cell viability. For antigen-presentation evaluation, the BMDCs were inoculated into a 12-well cell culture plate with 10^6^ cells well^-1^. After 6 h, BMDCs were incubated with OVA_257–264_ (SIINFEKL) peptide, An^hi^-Lipo, SIINFEKL/An^hi^-Lipo for 48 h (SIINFEKL, 1 μg mL^-1^). Then the BMDCs were collected, washed with PBS and incubation with Anti-SIINFEKL-H-2K^b^-PE antibodies at 4 ℃ for 1 h. After that, BMDCs were washed with PBS containing 2% FBS and detected by flow cytometry (BD Fortessa, USA).

For exploring the maturation of BMDCs, the BMDCs were inoculated into a 12-well cell culture plate with 10^6^ cells well^-1^. After 6 h, BMDCs were incubated with different charged liposome suspensions for 48 h (500 μg mL^-1^). Then BMDCs were collected, washed with PBS and incubation with Zombie UV™ Fixable Viability Kit to label dead cells. After washed with PBS containing 2 % FBS, BMDCs were incubated with anti-mouse CD11c-PerCP/Cyanine5.5, anti-mouse CD45-Brilliant Violet 510™, anti-mouse CD80-PE and anti-mouse CD86-Brilliant Violet 785™ for 30 min at 4 ℃. After that, BMDCs were washed with PBS containing 2 % FBS and detected by flow cytometry (BD Fortessa, USA).

**RNA-Sequencing assay**

The BMDCs were inoculated into a 6-well cell culture plate with 2×10^6^ cells well^-1^. After 6 h, BMDCs were incubated with PBS, OVA, An^hi^-Lipo, OVA/An^hi^-Lipo for 48 h (500 μg mL^-1^). Cells were collected for pre-detection by flow cytometry. The RNA libraries were sequenced on the illumina NovaseqTM 6000 platform by LC Bio Technology CO., Ltd (Hangzhou, China). Firstly, total RNA was extracted by VeZol Reagent and its total quantity, purity and integrity were tested by NanoDrop ND-1000 (NanoDrop, Wilmington, DE, USA) and Bioanalyzer 2100 (Agilent, CA, USA), separately. Invitrogen SuperScript^TM^ II Reverse Transcriptase (cat.1896649, CA, USA) was used for cDNA synthesis. Finally, we used illumina Novaseq^TM^ 6000 (LC Bio Technology CO., Ltd. Hangzhou, China) to double-end sequencing in PE150 mode according to standard procedures.

**Western Blot evaluation**

The BMDCs were inoculated into a 6-well cell culture plate with 2×10^6^ cells well^-1^. After 6 h, BMDCs were incubated with PBS, OVA, An^hi^-Lipo, OVA/An^hi^-Lipo for 48 h (500 μg mL^-1^). The BMDCs were collected, washed once with PBS, and 200 μL of RIPA lysate was added to each sample and blown for several times to make full contact between the lysate and the cells. Then the cells were centrifuged at 10000 g for 5 min, and the supernatant (protein sample) was carefully transferred into a new centrifuge tube and the protein concentration in different groups was determined by BCA kit (Beyotime, P0010S). Specifically, the total proteins were separated using Omni-EasyTM One-step Color PAGE Gel Rapid Preparation Kit (Epizyme PG212) and transferred into PVDF membranes. The membranes were combined with anti-GAPDH (Abcam, ab181602), MyD88 (Abcam, ab219413), TRAF6 (Abcam, ab33915), and NF-κB p65 (CST, 8242s), respectively. The primary antibody was incubated at 4°C overnight, and then combined with HRP-conjugated Affinipure Goat Anti-mouse lgG (H+L) (Proteintech, SA00001-1) at room temperature for 1 h. Protein bands were imaged using gel imaging system (Tanon-5200).

**Biodistribution of OVA-Cy5 and charged liposomes in *vivo***

C57BL/6 male mice of 8 weeks was randomly divided into four groups. OVA-Cy5, OVA-Cy5/Cat^hi^-Lipo, OVA-Cy5/Zw-Lipo and OVA-Cy5/An^hi^-Lipo were injected by subcutaneous injection at the root of tail, respectively (OVA-Cy5 1 mg mL^-1^, DiR 200 μg mL^-1^). The fluorescence distribution of Cy5 (Ex/Em: 750/780 nm) and DiR (Ex/Em: 646/662 nm) was detected by in vivo imaging system (IVIS) at 4, 8, 12, 24, 36 and 48 h post the injection. After that, the inguinal lymph nodes were collected and imaged ex vivo at 48 h. Furthermore, the immune cells were separated from the lymph nodes, stained and examined by flow cytometry. The uptake of Cy5 and DiR in DCs (CD45^+^CD11c^+^) was identified by flow cytometry.

**Immune response *in vivo***

BALB/c male mice of 8 weeks were randomly divided into seven groups and treated by different suspensions including PBS, OVA, OVA/Cat^hi^-Lipo, OVA/Cat^low^-Lipo, OVA/Zw-Lipo, OVA/An^low^-Lipo and OVA/An^hi^-Lipo were injected by subcutaneous injection on day 0, day 8 and day 16, respectively (OVA 5 mg kg^-1^; Liposome:OVA=10:3 (w/w); Liposome 16.67 mg kg^-1^). The mice were sacrificed on day 24, and the blood, spleen and lymph nodes were collected for examined the immune responses in vivo. The samples were processed into single cell suspensions and then stained with corresponding probe-labeled antibodies for examination. Specifically, mDCs were labeled by CD45^+^CD11c^+^CD80^+^CD86^+^. cDC1 was labeled by CD45^+^CD11c^+^B220^-^Lin^-^F4/80^-^CD172a^-^. cDC2 was labeled by CD45^+^CD11c^+^B220^-^Lin^-^F4/80^-^CD172a^+^. CD8^+^ T cell was labeled by CD45^+^CD3^+^CD8^+^ and IFN-γ^+^CD8^+^ T cell was labeled by CD45^+^CD3^+^CD8^+^IFN-γ^+^. Th1 was labeled by CD45^+^CD3^+^CD4^+^IFN-γ^+^. Th2 was labeled by CD45^+^CD3^+^CD4^+^IL-4^+^. Th17 was labeled by CD45^+^CD3^+^CD4^+^IL-17a^+^ and Treg was labeled by CD45^+^CD3^+^CD4^+^CD25^+^Foxp3^+^.

Sequentially, BALB/c mice with the same treatments were sacrificed on day 24. To evaluate hepatic and renal function, the blood of mice was collected and centrifuged for examination. The major organs including heart, liver, spleen, lung and kidney were collected for H&E staining to evaluate the biosafety of different treatments.

MC38-OVA tumor-bearing 8-week C57BL/6 male mice model was constructed for immunity analyzation. Mice were treated with PBA, OVA, Cat^hi^-Lipo, Zw-Lipo, An^hi^-Lipo, OVA/Cat^hi^-Lipo, OVA/Zw-Lipo, OVA/An^hi^-Lipo and MF59/OVA (OVA 5 mg kg^-1^; Liposome:OVA=10:3 (w/w); Liposome 16.67 mg kg^-1^; MF59 0.1mg per mouse). The mice in each group were treated with indicated treatments for three times every five days. Then MC38-OVA subcutaneous tumor model was constructed by subcutaneous injected with 1 million MC38-OVA tumor cells. The spleen and lymph nodes of mice in each group were collected 20 days later for immune cells investigation like in BALB/c mice.

**Anti-tumor study of charged liposomes in MC38-OVA tumor model**

For anti-tumor studies, C57BL/6 male mice of 8 weeks were randomly divided into eight groups. Then the mice were treated with PBS, OVA, Cat^hi^-Lipo, Zw-Lipo, An^hi^-Lipo, OVA/Cat^hi^-Lipo, OVA/Zw-Lipo, and OVA/An^hi^-Lipo via subcutaneous injection for three times at a time interval of 5 days (OVA 5 mg kg^-1^; Liposome:OVA=10:3 (w/w); Liposome 16.67 mg kg^-1^). The C57BL/6 mice were then inoculated subcutaneously with MC38-OVA tumor cells 2 days after the last treatment (10^6^ cells per mouse). Then the tumor volumes and body weight of mice were monitored during the anti-tumor study. The survival of mice was also monitored. During the anti-tumor study, the blood of mice was collected 2 days or 30 days post the inoculation of MC38-OVA cells, respectively. The lymphocytes in blood were separated and stained with OVA-epitope specific tetramer. Parallelly, the tumors in each group were collected and sectioned for H&E and immunofluorescent staining at desired time points.

**Statistical analysis**

Data analysis was carried out using t test (nonparametric tests) and one-way ANOVA (nonparametric tests). Data was presented as mean ± sd. **P<*0.05, ***P<*0.01, ****P<*0.001.

**Supporting Figures**

**
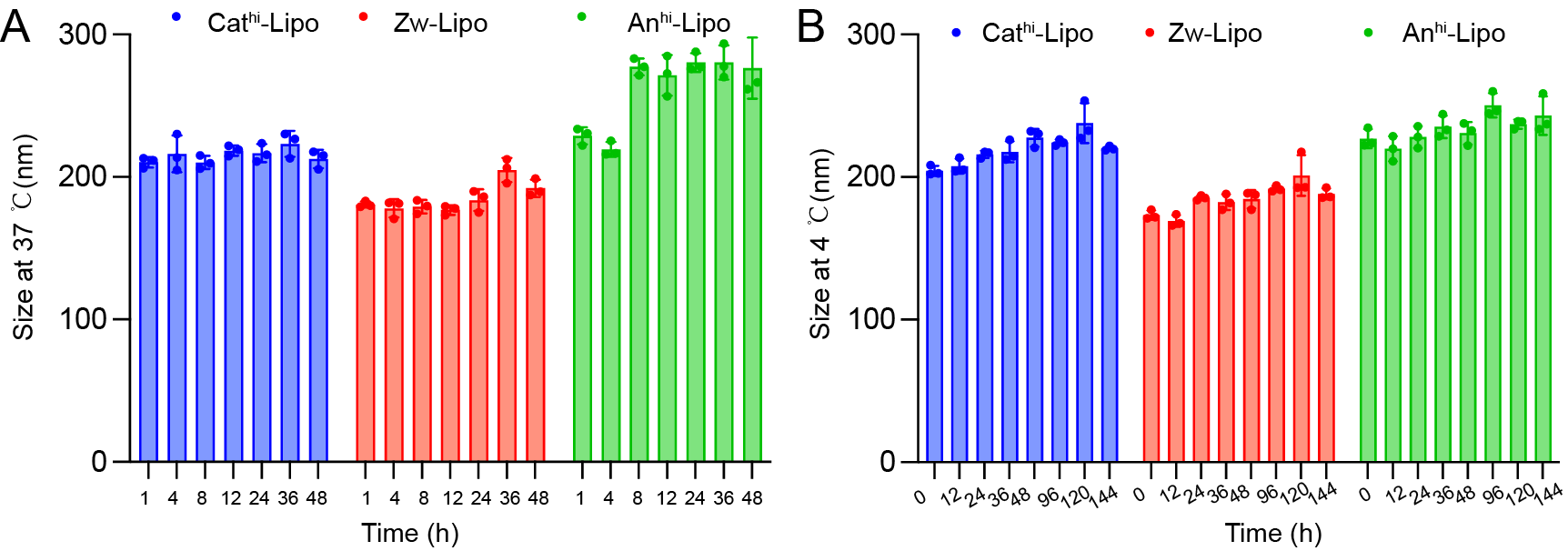
**

**Figure S1.** Stability of Cat^hi^-Lipo, Zw-Lipo and An^hi^-Lipo at the storage condition of 37 ℃ and 4 ℃, respectively (n=3).

**
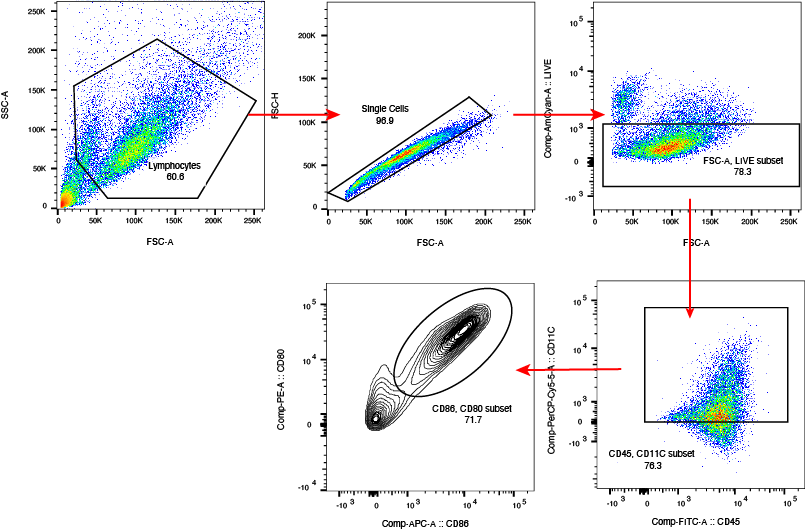
**

**Figure S2.** Gating strategy of mature BMDCs (CD45^+^CD11C^+^CD80^+^CD86^+^).


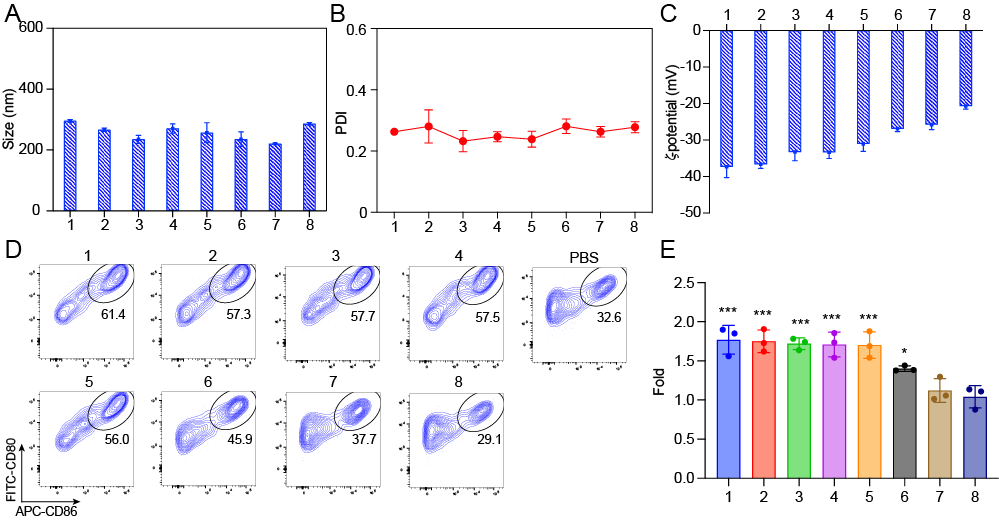


**Figure S3. Preparation of anionic liposomes and adjuvant effects investigation in vitro.** (**A**) The size of anionic liposomes of various lipid contents. (**B**) The ζ-potential of anionic liposomes of various lipid contents. (**C**) PDI of anionic liposomes of various lipid contents. (**D**) BMDCs were incubated with different antigen/adjuvant-free liposomes and the expression of CD80/CD86 was examined (n=3). (**E**) Increase fold of mature DCs after treated by charged liposomes when compared to PBS group. **P*<0.05, ****P*<0.001.


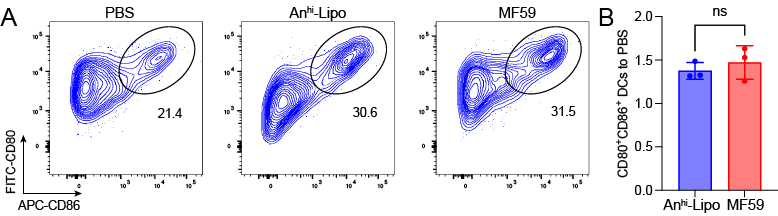


**Figure S4. Adjuvant effects of An^hi^-Lipo and MF59 in vitro.** (**A**) BMDCs were incubated with An^hi^-Lipo and MF59 for 24 h, respectively, and then the proportion of mDC (CD80^+^ CD86^+^) was measured by flow cytometry (n=3). (**B**) Increase fold of mature DCs after treated by An^hi^-Lipo and MF59 when compared to PBS group.


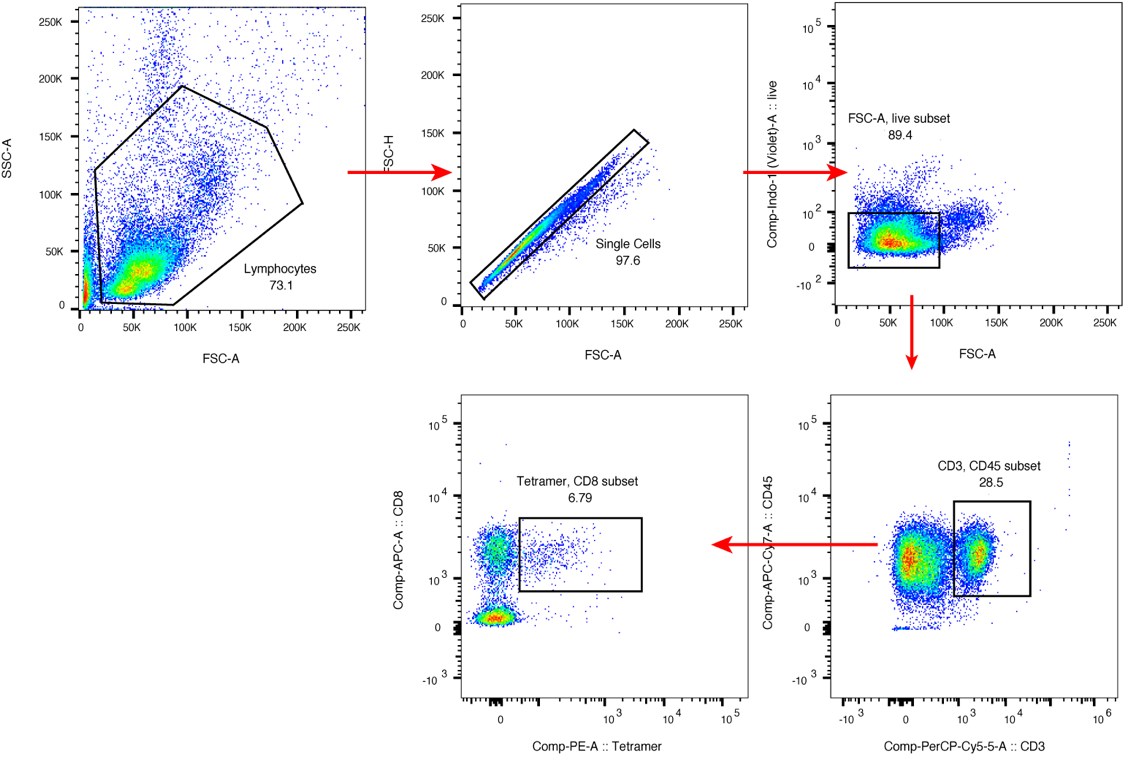


**Figure S5.** Gating strategy for detecting SIINFEKL-MHC I complex.

**
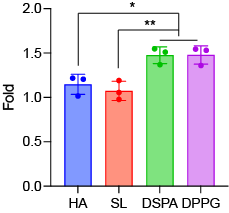
**

**Figure S6.** Increase fold of mature DCs after treated by charged liposomes when compared to PBS group (n=3). **P*<0.05, ***P*<0.01.

**
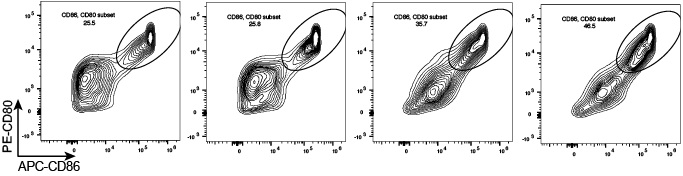
**

**Figure S7.** BMDCs were incubated with PBS, OVA, An^hi^-Lipo and OVA/An^hi^-Lipo. Proportion of mature BMDCs (CD45^+^CD11C^+^CD80^+^CD86^+^) was detected by flow cytometry (n=3).

**
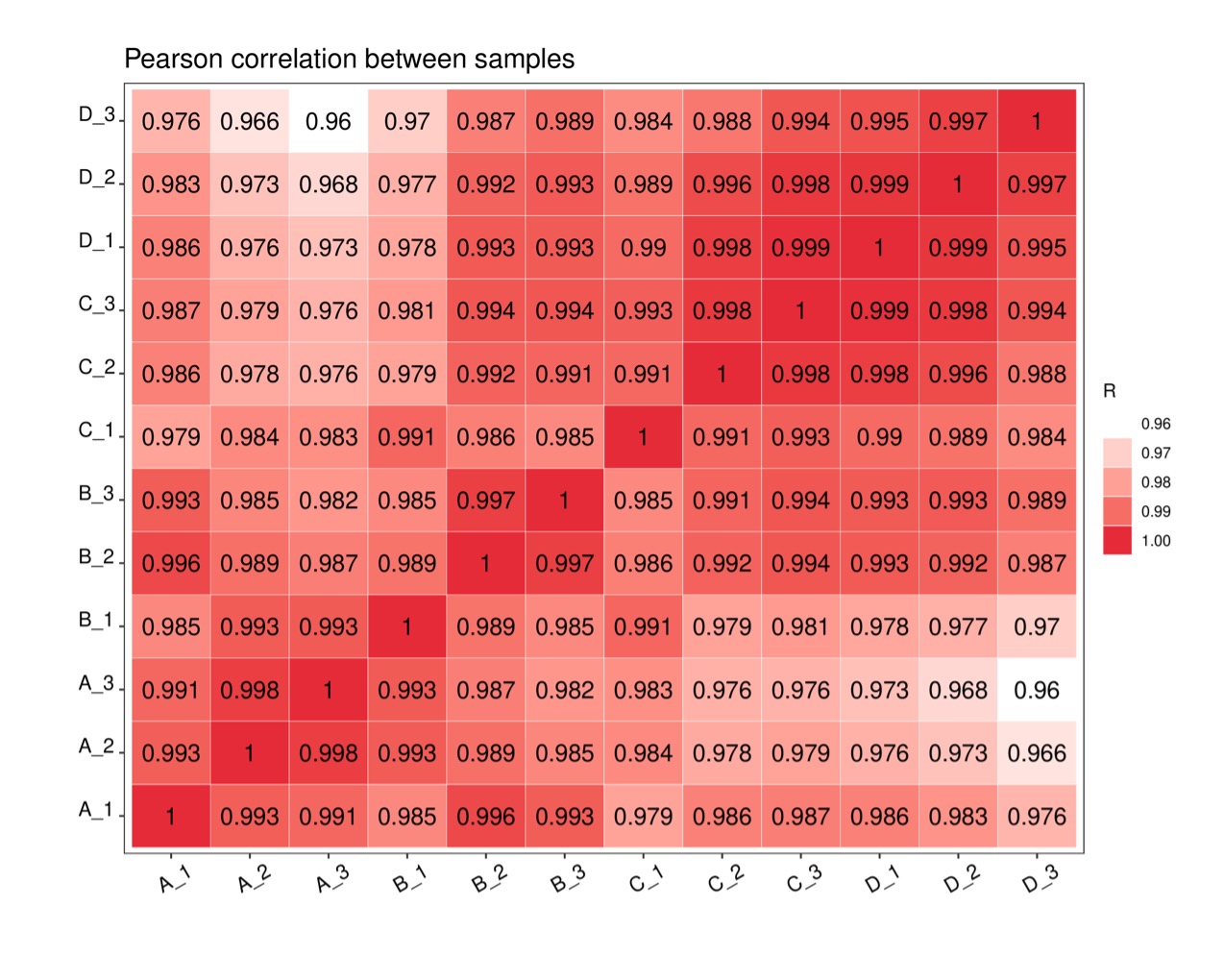
**

**Figure S8.** Principal Component Analysis (PCA) of gene samples from PBS, OVA, An^hi^-Lipo and OVA/An^hi^-Lipo. **
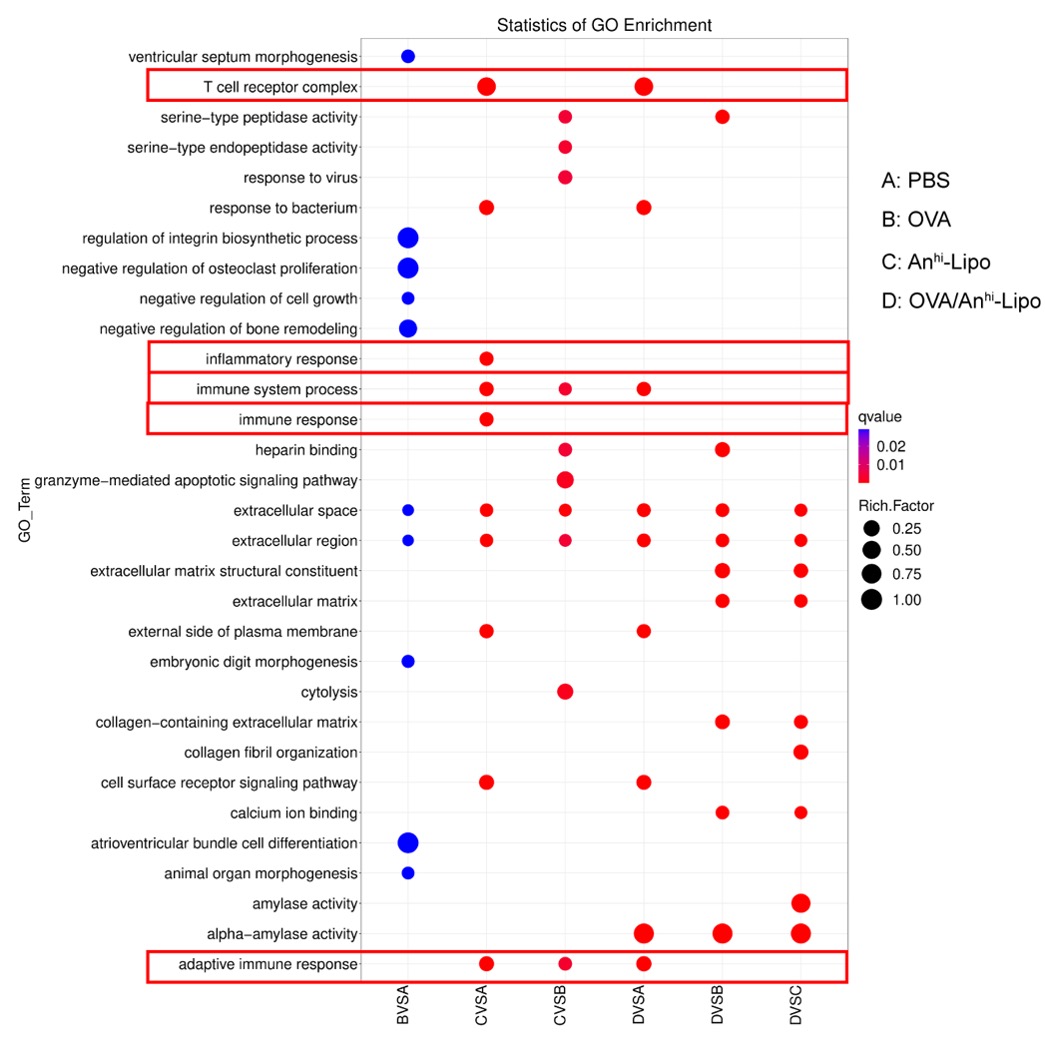
**

**Figure S9.** Statistics of GO enrichment among PBS, OVA, An^hi^-Lipo and OVA/An^hi^-Lipo.

**
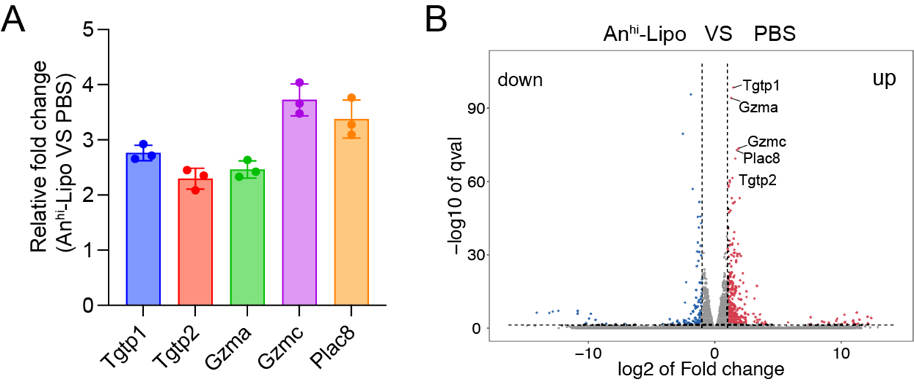
**

**Figure S10.** (**A**) When compared to PBS group, An^hi^-Lipo induced upregulation of Tgtp1, Tgtp2, Gzma, Gzmc and Plac8 in DCs. (**B**) Volcano maps showed the top differential genes between the compared two groups (n=3).

**
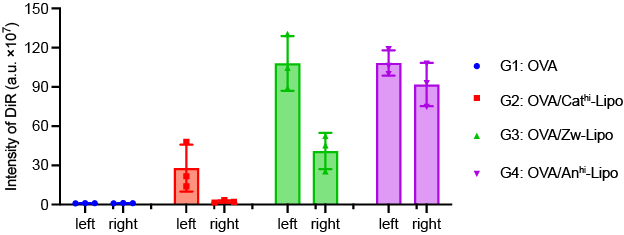
**

**Figure S11.** Fluorescence intensity of DiR in left and right inguinal lymph nodes were quantified (n=3).

**
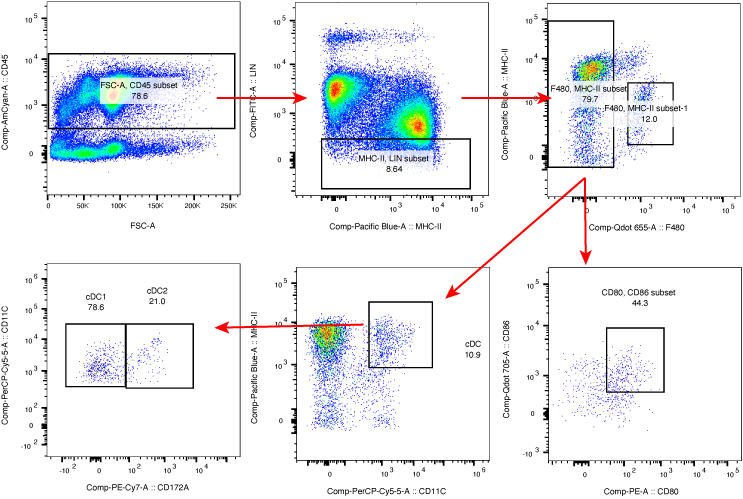
**

**Figure S12.** Gating strategy of cDC1 (CD45^+^CD11c^+^MHCII^+^F4/80^-^Lin^-^CD172a^-^), cDC2 (CD45^+^CD11c^+^MHCII^+^F4/80^-^Lin^-^CD172a^+^) and mDC (CD45^+^CD11c^+^MHCII^+^CD80^+^CD86^+^).

**
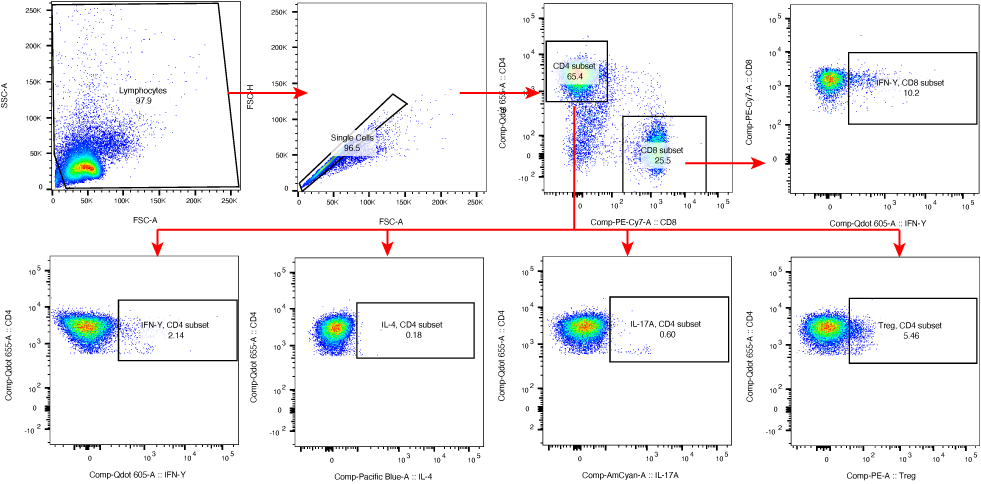
**

**Figure S13.** Gating strategy of CD4^+^ and CD8^+^ T cell.


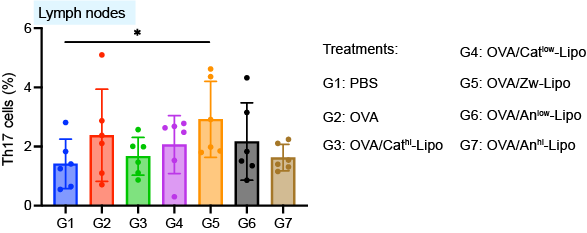


**Figure S14.** Th17 cells (CD45^+^CD3^+^CD4^+^IL-17A^+^) in lymph nodes were analyzed (n=6). **P*<0.05.

**
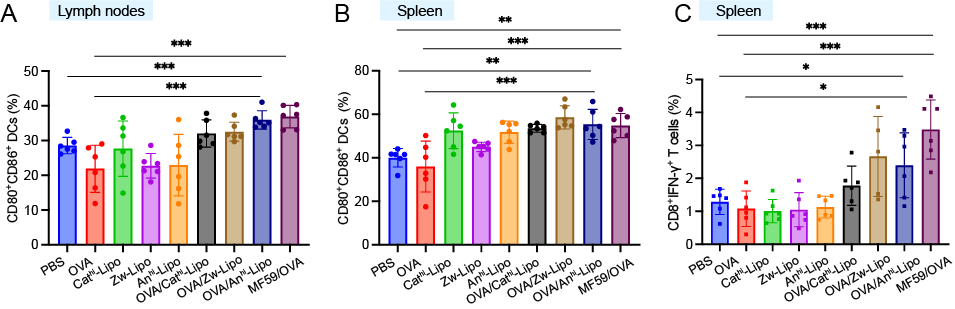
**

**Figure S15.** Th17 cells (CD45^+^CD3^+^CD4^+^IL-17A^+^) in lymph nodes were analyzed (n=6). **P<*0.05, ***P<*0.01, ****P<*0.001.

**
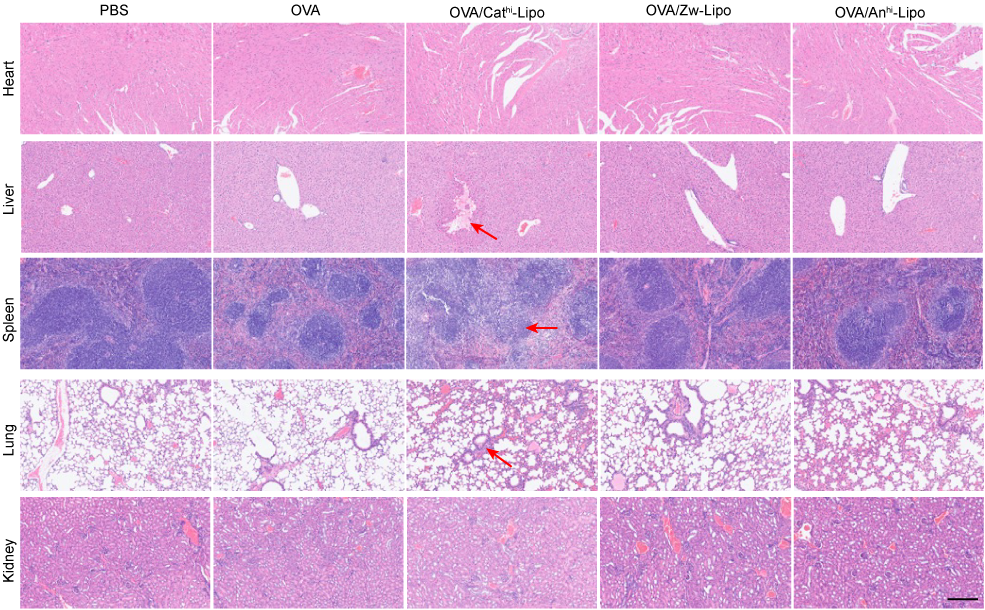
**

**Figure S16.** H&E staining of major organs (heart, liver, spleen, lung and kidney) of BALB/c mice (n=6).

**
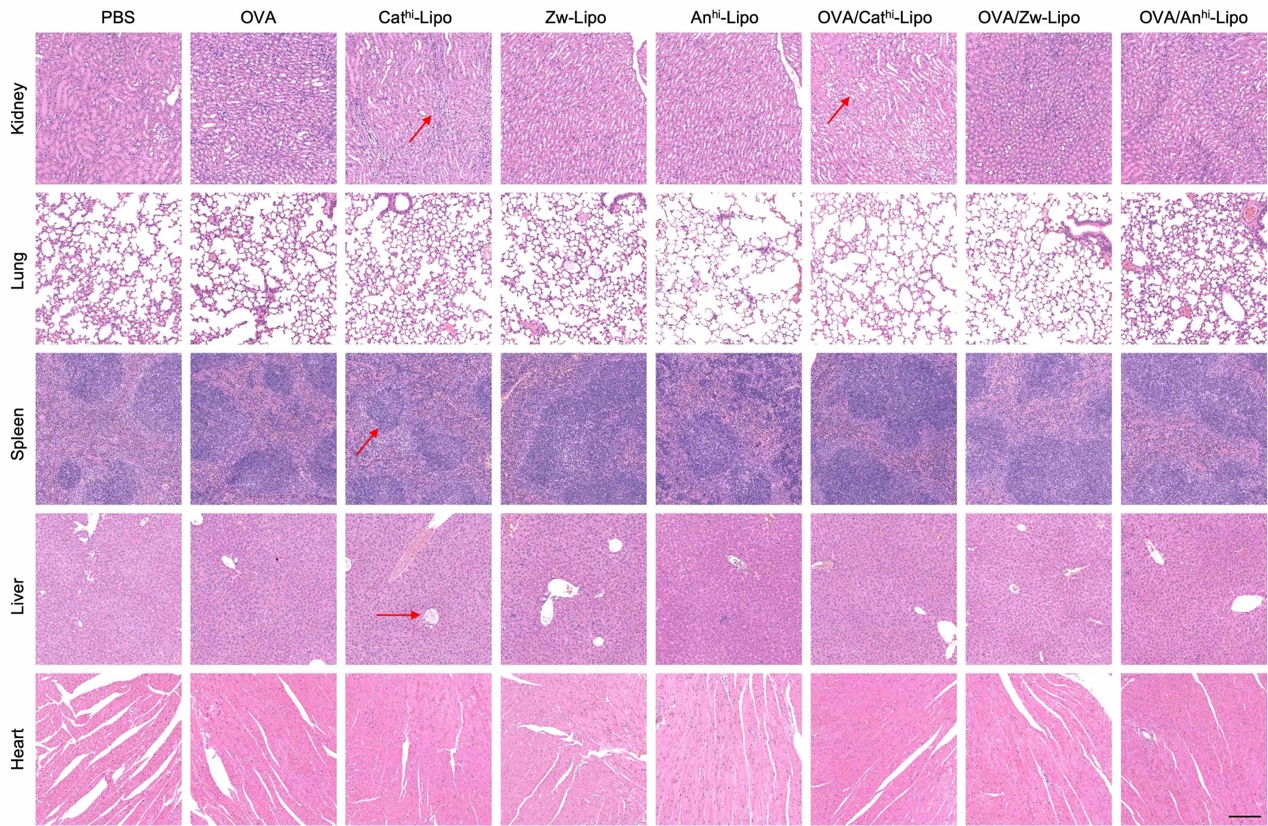
**

**Figure S17.** H&E staining of major organs (heart, liver, spleen, lung and kidney) of C57BL/6 mice (n=6).


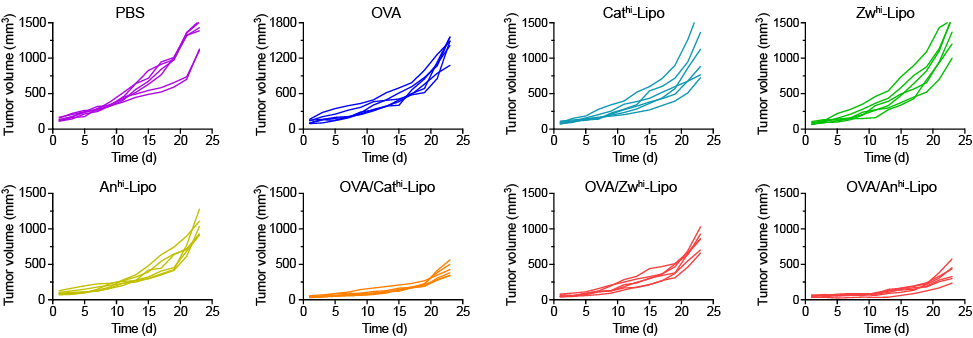


**Figure S18.** Individual tumor growth kinetics of mice in different groups (n=6).

**
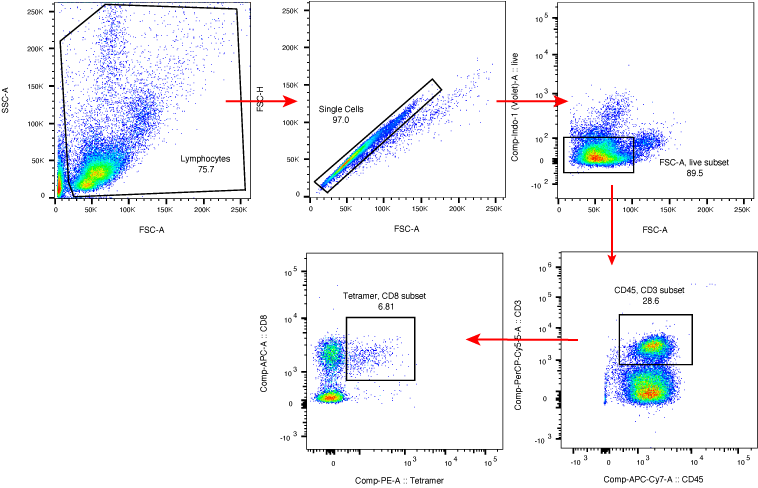
**

**Figure S19.** Gating strategy of OVA-specific CD8^+^ T cells.

**Supporting Tables**

**Table 1** Proportion of liposome containing DSPA prescriptions

| Prescription | DSPA | CHO-HP | DOPE | DMG-PEG_2000_ |
| --- | --- | --- | --- | --- |
| 1 | 55 | 40 | 4 | 1 |
| 2 | 50 | 40 | 9 | 1 |
| 3 | 45 | 40 | 14 | 1 |
| 4 | 40 | 40 | 19 | 1 |
| 5 | 35 | 40 | 24 | 1 |
| 6 | 30 | 40 | 29 | 1 |
| 7 | 25 | 40 | 34 | 1 |
| 8 | 20 | 40 | 39 | 1 |

Note: mol%
